# A Case of Acute Rheumatism Treated with Full Doses of Quinine

**Published:** 1853-10

**Authors:** W. M. Houston

**Affiliations:** Urbana, O.


From the Western Lancet,

*A Case of Acute Rheumatism Treated with full doses of Quinine,* By W. M. Houston, M.D., Urbana, O.

On Wednesday afternoon I was called to visit Miss G——, eight miles north-east of this place. On my arrival I obtained the following history of the case. The patient (16 years of age) was attacked on the previous Saturday with acute-rheumatism of a very severe character. On Sunday Dr. Johnson was called in, and for three days applied the usual remedies for rheumatism, viz.: bleeding, mercurial purgatives, opium, tartar emetic and colchicum, without any good effect; in fact the patient grew worse from day to day, until I first saw her, when she was unable to move hand or foot; as the least motion caused extreme suffering. Her pulse was one hundred, and strong, joints somewhat swollen and slightly red.

What course to pursue with a prospect of affording relief, after the failure of nearly all the anti-rheumatic remedies, was a question in regard to which physicians might well be excused for differing in their opinions, Dr. J. was in favor of further venesection, and taking the pulse as a guide, this measure seemed not only warranted but positively indicated. As, however, she had already been bled twice, I was fearful metastasis might result from the further loss of blood. I suggested the following treatment, (which, although seemingly empirical, was acceded to by Dr. J., as the usual remedies had most signally failed), viz.: 8 grs. quinine, 2 grs. opium, and 2 grs. ipecac every third hour, unless symptoms of narcotism should supervene; chloroform liniment to be applied to the joints; lemonade to be drank freely. I agreed to see the patient the next day, but being called in the night to attend an obstetrical case, (eight miles from town, in another direction), again until Sunday morning, when I learned that in the course of the first twelve hours she took 40 grs. quinine, 10 grs. opium, and 10 grs. ipecac; used 1 oz. liniment to the joints, but did not use the lemons. She rested well through the night, and in the morning felt greatly relieved. During Thursday and Friday she took about 20 grs. more of quinine, and enough colchicum to open the bowels. When I saw her on Saturday morning she was able to walk about the room—was entirely free from pain; pulse reduced to 70, of moderate force, and with the exception of a slight stiffness of the joints there was no evidence that the patient had ever had an attack of rheumatism.

